# Supplementary material for: Lycium barbarum Polysaccharides and Capsaicin Inhibit Oxidative Stress, Inflammatory Responses, and Pain Signaling in Rats with Dextran Sulfate Sodium-Induced Colitis
Source: Int J Mol Sci. 2022 Feb 22;23(5):2423. doi: 10.3390/ijms23052423 (PMC8910612; doi:10.3390/ijms23052423)
Supplement: Supplementary file 1 [file ijms-23-02423-s001.zip › ijms-1590711-supplementary.pdf]

Table S1. Definition of disease activity index score.

Score	Change of Body Weight	Fecal Consistency	Fecal Occult Blood
0	No changes or increases	Normal stiffness	No blood
1	Loss 1%-5%	Semi loose +	Occult blood +
2	Loss 5%-10%	Semi loose ++	Occult blood ++
3	Loss 10%-15%	Loose	Bloody feces +
4	Loss >15%	Diarrhea	Bloody feces ++

Table S2. Definition of histological scores.

Score	Abnormality of Mucosal Architecture	Extent of Inflammation	Erosion or Ulceration	Epithelial Regeneration	Percent of Involvement
0	No changes	No infiltration	No ulceration	Complete structure	-
1	Structure slightly regional abnormality, but not affect lamina propria	Slight and regional inflammation (<10%)	Slight and regional, did not penetrate through lamina propria	Most tissues were regenerated	1%-25%
2	Slight abnormality of structure and glands, extension of cystic lesion	Slight inflammation (10%-25%)	Obvious damage	Regeneration of gland structure	26%-50%
3	Moderate abnormality, multiple abnormal lesion	Moderate inflammation, neutrophil infiltrate in submucosa	Moderate ulceration	Incomplete structure of epithelial layer	51%-75%
4	Severe lesion, loss of glands and epithelial structure	Severe inflammation, neutrophil infiltrate from mucosa to serosa layer	Severe damage and formation of granulation tissue	No regeneration of tissue	76%-100%
